# P-795. Unravelling the Enigma: Evaluating False Positive and Invalid Results in Urinary TB LAM Test among HIV Patients with Opportunistic Infections

**DOI:** 10.1093/ofid/ofae631.987

**Published:** 2025-01-29

**Authors:** Shivang Sharma, Deepak Kumar

**Affiliations:** Zydus Hospital, Ahmedabad , Gujarat, India; All India Institute of Medical Sciences, Jodhpur (India), Jodhpur, Rajasthan, India

## Abstract

**Background:**

Various studies suggested that successful implementation of urine Lateral Flow –Lipoarabinomannan (LF LAM) testing could greatly improve TB diagnosis and treatment in HIV patients.Studies from India are scarce regarding urine LF-LAM testing. One should be careful while interpreting the positive test as this test can be positive in NTM, or nocardiasis. This case study highlights the false positive test result of LF-LAM testing and its impact on clinical decision-making in HIV TB co-infection.

**Methods:**

In,this prospective observational hospital based study total 95 adult patients had been included consecutively regardless of their presenting symptoms or CD4 count.During this study, all recruited patients were underwent for relevant investigation for diagnosing opportunistic infections including TB and simultaneously urine LF-LAM assay tests were performed during hospitalisation.Urine LF- LAM testing had been conducted using the Determine Ag assay and interpreted using the 4-grade scale provided by the manufacturer.

**Results:**

Out of total 95 patients, 31 were diagnosed with TB, and 25 tested positive for LAM. However, among the 25 LAM-positive patients, five were found to have false-positive results, while two patients received invalid results. Among the six cases of false-positive TB LAM results, three patients had confirmed NTM (Non-tuberculous mycobacterial) infections, one case was culture-positive for nocardiosis, and another had Cryptococcal infection. One case involved urosepsis with Diabetic Ketoacidosis. All five cases were negative for ZN stain, GeneXpert, and TB culture, and no ATT was administered. Tragically, two patients among these five expired. Additionally, both patients with invalid results had chronic kidney disease.

**Conclusion:**

There can be false-positive TB LAM results with several clinical conditions like NTM infections,Nocardiosis,cryptococosis.These clinical conditions are close differential diagnosis of TB and requires invasive or advance testing methods. While urinary TB LAM testing demonstrates promising sensitivity in diagnosing tuberculosis among hospitalized HIV patients, the occurrence of false-positive results and invalid result outcomes alert clinicians and one should exercise caution in interpreting TB LAM results.

**Disclosures:**

**All Authors**: No reported disclosures

